# Segmentation of 3D OCT Images of Human Skin Using Neural Networks with U-Net Architecture

**DOI:** 10.17691/stm2025.17.1.01

**Published:** 2025-02-28

**Authors:** V.A. Shishkova, N.V. Gromov, A.M. Mironycheva, M.Yu. Kirillin

**Affiliations:** Junior Researcher; A.V. Gaponov-Grekhov Institute of Applied Physics of the Russian Academy of Sciences, 46 Ulyanov St., Nizhny Novgorod, 603950; Junior Researcher; National Research Lobachevsky State University of Nizhny Novgorod, 23 Prospekt Gagarina, Nizhny Novgorod, 603022, Russia; Junior Researcher; A.V. Gaponov-Grekhov Institute of Applied Physics of the Russian Academy of Sciences, 46 Ulyanov St., Nizhny Novgorod, 603950 Assistant, Department of Skin and Venereal Diseases; Privolzhsky Research Medical University, 10/1 Minin and Pozharsky Square, Nizhny Novgorod, 603005, Russia; PhD, Senior Researcher; A.V. Gaponov-Grekhov Institute of Applied Physics of the Russian Academy of Sciences, 46 Ulyanov St., Nizhny Novgorod, 603950

**Keywords:** optical coherence tomography, thick skin, stratum corneum, epidermis, convolutional neural networks, U-Net architecture

## Abstract

**The aim of the study** is a comparative analysis of algorithms for segmentation of three-dimensional OCT images of human skin using neural networks based on U-Net architecture when training the model on two-dimensional and three-dimensional data.

**Materials and Methods:**

Two U-Net-based network architectures for segmentation of 3D OCT skin images are proposed in this work, in which 2D and 3D blocks of 3D images serve as input data. Training was performed on thick skin OCT images acquired from 7 healthy volunteers. For training, the OCT images were semi-automatically segmented by experts in OCT and dermatology. The Sørensen–Dice coefficient, which was calculated from the segmentation results of images that did not participate in the training of the networks, was used to assess the quality of segmentation. Additional testing of the networks’ capabilities in determining skin layer thicknesses was performed on an independent dataset from 8 healthy volunteers.

**Results:**

In evaluating the segmentation quality, the values of the Sørensen–Dice coefficient for the upper stratum corneum, ordered stratum corneum, epidermal cellular layer, and dermis were 0.90, 0.94, 0.89, and 0.99, respectively, for training on two-dimensional data and 0.89, 0.94, 0.87, and 0.98 for training on three-dimensional data. The values obtained for the dermis are in good agreement with the results of other works using networks based on the U-Net architecture. The thicknesses of the ordered stratum corneum and epidermal cellular layer were 153±24 and 137±17 μm, respectively, when the network was trained on two-dimensional data and 163±19 and 137±20 μm when trained on three-dimensional data.

**Conclusion:**

Neural networks based on U-Net architecture allow segmentation of skin layers on OCT images with high accuracy, which makes these networks promising for obtaining valuable diagnostic information in dermatology and cosmetology, e.g., for estimating the thickness of skin layers.

## Introduction

Optical coherence tomography (OCT) is a modern technique for optical noninvasive visualization of biological tissues based on the principles of lowcoherence interferometry providing spatial resolution up to microns [1, 2]. OCT has been widely used clinically in ophthalmology, but has potential in noninvasive visualization of the internal structure of skin and mucous membranes. The advantage of OCT employment in ophthalmology originates from weak light scattering in the eye tissues, while stronger scattering in skin and mucous membranes limits practical probing depth to values of the order of 1–2 mm [3]. Nevertheless, such probing depth allows to analyze the main characteristics of skin structural layers: stratum corneum, epidermis, dermis.

Approaches to segmentation of ophthalmologic OCT images have been proposed earlier (see, for example, [4]), including for three-dimensional diagnostic images [5]. Weak scattering of probing radiation in structural layers of eye tissues provides rather high contrast of layer boundaries in OCT images, which makes the problem of layer segmentation relatively simple. In highly scattering media, including skin, several typical features can be identified, such as pronounced attenuation of OCT signal with probing depth and relatively low contrast of structural layers, comparable in some cases with the contrast of speckle structure of OCT image [6]. Manual segmentation of two-dimensional OCT images is resource-consuming, and systemic segmentation of three-dimensional OCT images without tools that automate this process looks very difficult. Thus, effective application of OCT in dermatology requires development of tools capable to quickly and qualitatively extract necessary diagnostic information from the obtained three-dimensional skin images. This will contribute to a wider use of OCT in clinical dermatology.

Attempts to automate the segmentation of OCT images of skin have been made for a long time. For example, in 2006 a group of scientists performed segmentation of three-dimensional skin images [7] to determine the upper boundary of the skin, as well as the separation of the epidermis from the dermis; then hair follicles were highlighted on the images. The proposed algorithm allowed estimation of the average epidermal thickness. The algorithm used a sequence of median filters applied to the surfaces of intensity peaks, followed by approximation of the layer boundary by a polynomial function. Thus, this algorithm engages manually selected empirical parameters. In the paper [8], a segmentation method based on support vector machine (SVM) classification of statistical speckle distribution was proposed. Another approach that has been applied by several research groups is the use of graphs. For example, paper [9] proposed a method for detecting the upper boundary of skin and lower boundary of epidermis, which includes several steps: preprocessing based on weighted least squares method, detecting the upper boundary of skin using graph, and detecting the boundary between epidermis and dermis based on local integral projection. Graph theory has also been applied to automatically detect the skin surface and the boundary between epidermis and dermis in OCT images of skin in studies [10, 11]. Note that the above-mentioned works were based on classical image processing methods.

In 2015, a group of scientists from the University of Freiburg developed a U-Net architecture specifically for medical image segmentation [12]. It was created taking into account the fact that the sample size of training images for medical problems can be significantly limited, and the boundaries of the selected areas are not always obvious due to possible noise in images obtained with custom medical equipment.

The U-Net architecture and other convolutional neural network architectures then found widespread use in ophthalmology and dermatology. The articles [4, 13] discuss the application of U-Net for segmentation of retinal OCT images. However, in transparent media the contrast of boundaries is much higher compared to highly scattering media, so these methods require further development when adapting them to the problem of segmentation of OCT images of skin.

In 2018, convolutional U-Net was used to determine the boundary between epidermis and dermis [14]. In 2019, a modified U-Net (densely connected convolutions added) was used to analyze OCT images of laboratory animal tissues, namely for segmentation of skin, subcutaneous fat layer, fascial-muscular layer and tattoos used as reference marks [15]. Other convolutional network architectures such as ResNet18 [16] or CE-Net [17], which combine ResNet and U-Net, are also used for segmentation of OCT images of laboratory animal tissues.

A number of studies on the application of U-Net architecture in the development of algorithms for segmentation of diagnostic skin OCT images should be emphasized. The paper [18] presents an approach to segmentation of the epidermal layer together with follicular structures in OCT images of healthy volunteers’ skin using a convolutional neural network based on the U-Net architecture with post-processing consisting in image filtering. The paper [19] presents an approach to segmentation of skin images obtained with high-frequency ultrasound (the features of OCT and ultrasound images are similar) with preprocessing and subsequent application of U-Net. In the work [20], the stratum corneum, epidermis, and dermis were distinguished in OCT images of human skin with the help of U-Net architecture, which training was carried out only on images of healthy skin areas, and the algorithm was also used for processing images of skin with damage, such as scar tissue from laser treatment or tumor. As part of an experiment on laboratory mice [21], the U-Net architecture was also used to segment OCT images of laser-damaged skin areas. In [22], a U-Net-based segmentation model pre-trained on rodent skin OCT images was proposed for additional training on human skin data. The authors claim that with such an approach a single 2D segmented image from a 3D volume is sufficient to accurately segment the entire 3D image for a single patient.

Thus, recent works on OCT image segmentation emphasize the use of U-Net architecture or similar convolutional networks, which allows us to justify the preference of such architecture when selecting a model for segmentation. It should be noted that in almost all of the cited papers, model training was performed on the basis of preprocessed two-dimensional data (B-scans), including cases when the algorithms were subsequently applied for segmentation of three-dimensional data. Such analysis was predominantly performed for each individual B-scan from the entire dataset. However, there are studies that show that the use of volumetric information can improve segmentation prediction results [23].

**The purpose of this work** is a comparative analysis of algorithms for segmentation of three-dimensional OCT images of human skin using the U-Net architecture when training a model on two-dimensional and three-dimensional data. The study was conducted on a sample of three-dimensional images of thick skin (localized of fingers). Both approaches are compared in terms of the quality of image segmentation in the problem of delineating the boundaries of four structural layers: the upper layers of stratum corneum, the ordered stratum corneum, the cellular layer of epidermis, and the dermis.

## Materials and Methods

### System for optical coherence tomography

**Figure 1.**
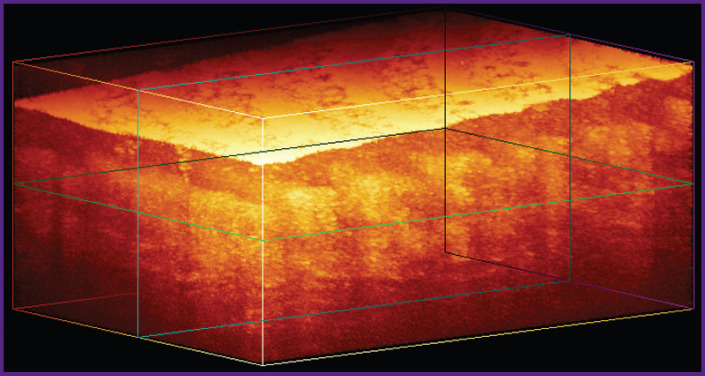
Three-dimensional OCT image of human thick skin obtained with OCT-1300E device

To obtain images of human skin in the study we used OCT-1300E device (IAP RAS, Biomedtech LLC, Russia) with a central wavelength of 1300 nm equipped with a contact fiber-optic probe. The setup allows obtaining three-dimensional OCT images with axial (depth) spatial resolution of 15 μm. The output data *I*(*x*, *y*, *z*) is an array of 256×512×512 elements (Figure 1), where each element corresponds to an OCT signal from the corresponding voxel in relative units. The physical dimensions of the visualized volume are 1.2×3.0×3.0 mm. A typical image of thick human skin obtained with OCT-1300E is presented in Figure 1.

### Dataset

In this study, a set of labeled OCT images of thick skin obtained from 7 healthy volunteers (21– 45 years old; 3 males, 4 females) was used to train convolutional networks. All images were taken from the pad of the distal phalanx of the index finger. A set of 16 thick skin images acquired from 8 healthy volunteers (21–45 years old; 3 males, 5 females) was additionally used to test the ability of the networks in determining the thicknesses of the structural layers of skin. The study was approved by the local ethical committee of Privolzhsky Research Medical University (protocol No.17 of October 11, 2019). Due to the peculiarities of the fiber optic probe of the OCT device, the image texture in the border regions (left and right borders of a B-scan) may be distorted compared to the central part of the image, so a central part of 256×512×256 voxels in each array was left for study from each 3D dataset.

### Primary segmentation of OCT images

**Figure 2.**
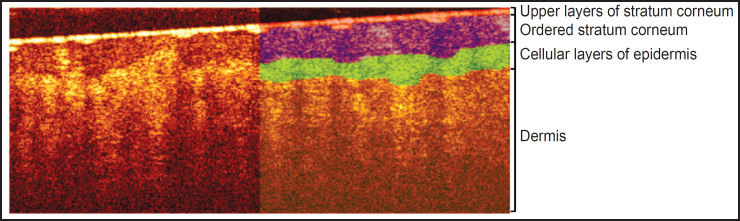
Typical segmented two-dimensional OCT image of thick skin The image size is 3.0×1.2 mm

The layers of thick skin can be most clearly identified in a twodimensional OCT image (B-scan). A typical OCT B-scan of human thick skin is shown in Figure 2. The left side is the original OCT image, while the right side shows a labeled image in which four structural layers are highlighted. The feature of thick human skin is thick stratum corneum, which can be divided into two layers [24]: a thin upper layer of disordered scales (in OCT images it is manifested by a thin layer with high signal intensity, similar to the stratum corneum of thin skin) and a thick layer of ordered scales (in OCT image it is manifested by a layer with reduced signal level). Below the stratum corneum cellular layer of epidermis is situated, which is characterized by a higher signal intensity compared to the ordered scales of the stratum corneum, below the epidermis the dermis is located, characterized by a lower OCT signal level compared to the cellular layer of epidermis. The lower border of the dermis cannot be detected in an OCT image, since the imaging depth of the employed OCT system is less than the full thickness of the thick skin.

The initial data labeling for training networks based on the U-Net architecture was performed using a semiautomatic approach and then verified by OCT specialists and dermatologists. Semi-automatic segmentation was performed by a method similar in principle to that proposed in [12], which is based on determining the average signal level (mean) and its standard deviation (std) in each of the layers *i*={2, ..., 5} (*i*=1 corresponds to the space inside the probe above the skin surface in the OCT image). To implement this approach, in each layer, a cuboid region *M_i_* was pre-selected, which was guaranteed to belong to the given layer, i.e., visually did not include boundary voxels. Then, for each of the layers, the OCT signal level, presumably corresponding to the upper boundary of the layer, was determined based on the statistics of signal distribution in the selected area *M_i_*:

Iit=meanMi(I)αistdMi(I),

where α_*i*_ is an empirically selected parameter. Based on the value of $I_{i}^{t}$, the surface of the upper boundary of a certain layer *i* was detected, and a median filter with a window size of *w_i_*=11 voxels was then applied to its longitudinal coordinate (*z*). The obtained surface was visually estimated by experts for compliance with the real boundary. In case of unsatisfactory results, the parameters α_*i*_ and *w_i_* were re-selected, and then the procedure was repeated. After finding the surface corresponding to the upper boundary of layer *i*=2 (the upper layer of stratum corneum), a median filter was applied to the signal level of the image area under this boundary to reduce speckle noise. Next, the same procedure for determining layer boundaries was performed for the underlying layers (*i>*2).

This method has a significant disadvantage because it requires empirical selection of parameters at each step and visual control of the labeling quality by a specialist. However, it should be noted that it is significantly faster than fully manual data labeling when labeling threedimensional datasets.

### Application of U-Net architecture for OCT skin image segmentation

The main task of the neural network in the considered problem is to attribute a class label to each voxel of the OCT image. The choice is restricted to five classes: background (space above the skin surface), upper layers of the stratum corneum, ordered stratum corneum, cellular layer and dermis. Accordingly, the input data for the neural network is a three-dimensional array of OCT signal values *I*(*x*, *y*, *z*) and the corresponding three-dimensional array of labels of class *K*(*x*, *y*, *z*), where each voxel is mapped to an integer value varying from 1 to 5. This study compares the application of two convolutional neural networks based on the U-Net architecture using 2D or 3D data for training.

The basic idea of the network architecture is to supplement the usual contracting path (the left part of the letter “U”) with an expansive path, decoder (the right part of the letter “U”). These layers increase the output resolution. To preserve localization information, the layers of the contracting path and expansive path of the network are connected by skip connections. When solving the small data problem, the original paper [11] uses data augmentation, applying elastic deformations to the existing labeled images. This allows the network to learn invariance to such deformations without having to see the transformation data in the labeled image dataset.

**Figure 3.**
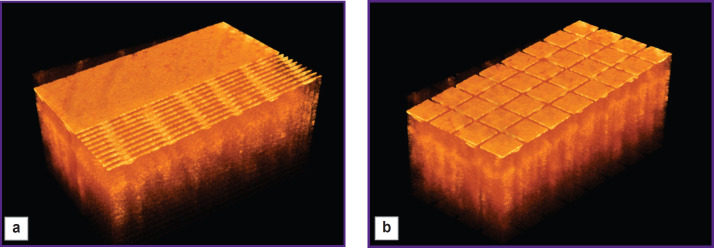
Splitting of 3D OCT image into blocks for training: (a) two-dimensional blocks and (b) three-dimensional blocks

To provide a sufficient training sample size, the obtained three-dimensional data sets are divided into blocks, the set of which is used to train the neural network. This study compares two types of block splitting. In one case, B-scans of 256×512×1 voxels (two-dimensional data) represent the block, while in the other case, three-dimensional blocks of 256×64×64 voxels are used. The selection of blocks for the two considered cases is shown in Figure 3.

**Figure 4.**
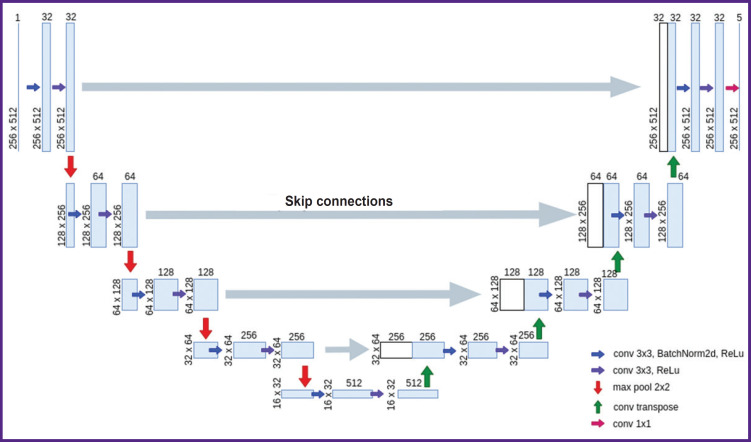
Network architecture for OCT image segmentation with training on two-dimensional data (2D U-Net)

***The architecture of the network for training on two-dimensional data (2D U-Net)*** repeats the standard U-Net architecture, which is described in the original article [11], with minor changes (Figure 4). The network consists of two parts: an encoder (contracting path, left side) and a decoder (expansive path, right side). The encoder branch has 5 stages and is responsible for extracting multi-scale features of the input image. The decoder branch also includes 5 stages and is required to upsample the feature map obtained after the encoder, and due to the skip connections in this branch it is possible to recover the exact localization of the features obtained in the encoder. Each encoder stage consists of a 3×3 convolution with a 1 voxel padding of the original image, followed by patch normalization and a nonlinear ReLu activation function. Further another similar convolution and ReLu activation are performed. A 2×2 maxpool operator is then applied to the resulting feature maps, which reduces the spatial dimensions, thereby compressing the information and allowing the number of feature maps to increase. The 1^st^, 2^nd^, 3^rd^, 4^th^, and 5^th^ stages of encoding generate 32, 64, 128, 256, 512 features, respectively. In the decoder branch, each stage includes a connection to the corresponding layer from the symmetric encoder, followed by a 3×3 convolution, batch normalization, a nonlinear ReLU activation function, another 3×3 convolution, and a final ReLU activation. For the new decoder stage, a transposed convolutional layer is applied to the feature maps in order to increase the discretization of the feature maps. The last block of the decoder consists of a convolution layer with a 1×1 kernel. Thus, to each voxel five confidence levels of belonging to each of the classes are assigned, after which the voxel is assigned to the class corresponding to the highest confidence level.

When using the 2D U-Net model to segment a 3D image, it was divided into blocks of 256×512×1 voxels. For each such block, segmentation was performed, after which the obtained data were combined into a 3D array.

**Figure 5.**
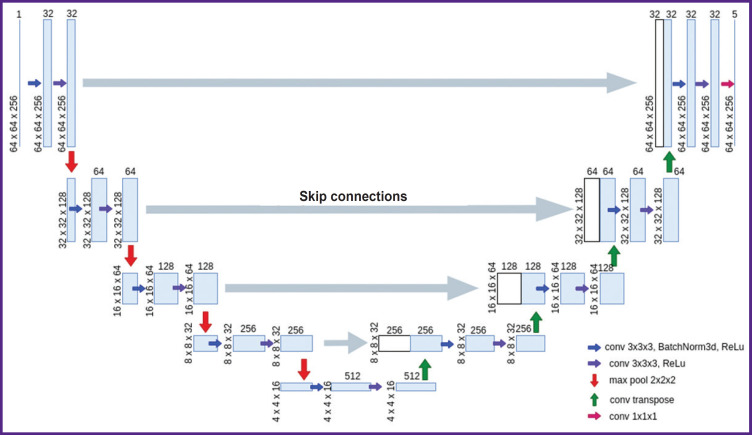
Network architecture for OCT image segmentation with training on three-dimensional data (3D U-Net)

***The architecture of the network for training on three-dimensional data (3D U-Net)*** follows the architecture described above with the difference that the input is represented by a block of size 256×64×64 voxels (Figure 5), and 2D operations are replaced by their 3D analogues (e.g., 2D convolutions are replaced by 3D convolutions). To segment the full 3D OCT image with the 3D U-Net model, the image was split into blocks with intersections of size 256×64×64 voxels, for each of which five 3D confidence degree maps of belonging to each class were computed independently. For the voxels belonging to the block intersection region, the degree of confidence of belonging to each class was defined as the sum of the values for each of the blocks weighted by a Gaussian function with a radius of 16 voxels as a function of the distance to the center of the block. This procedure was followed by assigning a class value to each voxel according to the maximum confidence value, similar to the 2D U-Net application. This allowed us to get rid of the peculiarities occurring at block boundaries.

### Training of networks with U-Net architecture

The available dataset consisted of 7 3D images of size of 256×512×256 voxels from 7 different volunteers. In order to avoid overtraining and incorrect values during quality assessment on dependent data, two 3D arrays out of the seven were kept for the test sample and were not used in the training process. Next, a cross-validation method, namely leave-one-out cross-validation, was applied, where one 3D image from a particular volunteer is considered as an object. From the five models built, the one that demonstrated the best results during validation was selected. This model was applied to the test sample.

The 2D U-Net network received two-dimensional images of size 256×512×1 as input. Such images were obtained from 3D images as slices along the axis with a dimensionality of 512 voxels, which provided 256 blocks for training for each OCT image. The 3D U-Net network received three-dimensional images of size 256×64×64 as input. To obtain them, the 3D image was divided without intersections into such blocks, which provided 32 blocks for training for each 3D OCT image.

The loss function, cross-entropy, and the ADAM (Adaptive Moment Estimation) optimizer were used for training. These are the most well-proven loss function and optimizer in such tasks. The learning rate coefficient was set to 0.001. The coefficients used to compute the moving averages of the gradient and its square were set as betas=(0.9, 0.98).

### Testing process

The Sørensen–Dice coefficient (DSC) was used in the quality assessment. Let *K^true^* (*x*, *y*, *z*) be the true array of class labels and *K^segm^* (*x*, *y*, *z*) be the array of class labels obtained by applying the neural network. Then the Sørensen–Dice coefficient is the ratio of the doubled number of voxels of a certain class matched in the arrays *K^true^* (*x*, *y*, *z*) and *K^segm^* (*x*, *y*, *z*) to the sum of the number of voxels of these classes in each of the arrays:

DSCi=2n(Ktrue(x,y,z)=Ksegm(x,y,z)=i)n(Ktrue(x,y,z)=i)=n(Ksegm(x,y,z)=i),

where *i* is the class index value; the function *n*(...) returns the number of voxels for which the condition in brackets is satisfied. The DSC coefficient takes values from 0 to 1, where a value of 1 corresponds to the case of a perfect match between the labeled and predicted masks. This is a metric that is widely used to evaluate the quality of segmentation algorithms. The metric was computed for 3D masks for both models.

## Results

**Figure 6.**
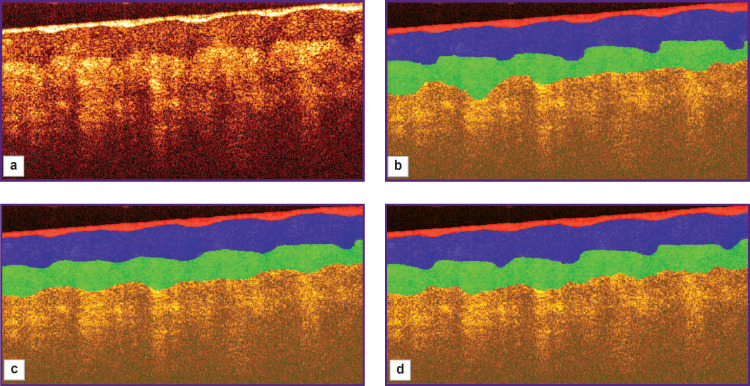
Segmentation of an OCT image of thick skin: (a) original; (b) semi-automatic segmentation; (c) segmentation by 2D U-Net model; (d) segmentation by 3D U-Net model

An example of segmentation of structural layers in a two-dimensional OCT image is shown in Figure 6. Figure 6 (a) shows the original OCT image, Figure 6 (b) represents the segmentation obtained in semiautomated mode under expert supervision, Figure 6 (c) shows the segmentation results using 2D U-Net, and Figure 6 (d) shows the result of processing with 3D U-Net.

**Figure 7.**
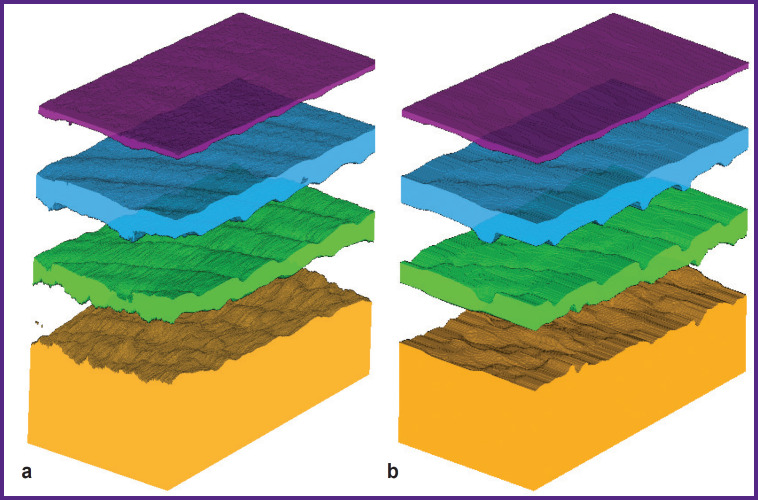
Result of 3D OCT image segmentation using 2D U-Net (a) and 3D U-Net (b)

Figure 7 shows a visualization of the results of applying 2D U-Net and 3D U-Net to the entire 3D array.

**Figure 8.**
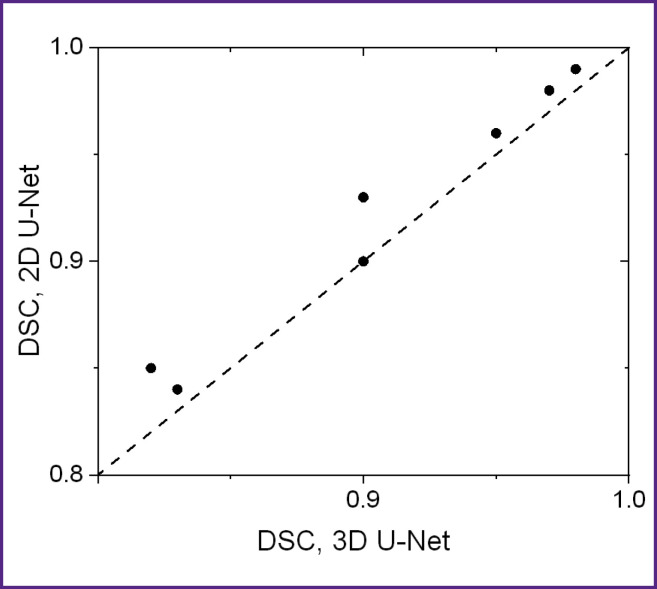
Comparison of DSC coefficients for 2D U-Net and 3D U-Net models for layer segmentation on 3D OCT images from the test sample

To numerically characterize the quality of segmentation of 3D OCT images by 2D U-Net and 3D U-Net networks, segmentation was performed for two marked OCT images that were not included in the training sample. Figure 8 shows a diagram of the correspondence of DSC coefficient values for different image layers from the test sample.

Table 1 shows the averaged values of the Sørensen– Dice coefficients for each layer in the 3D images of the test sample.

**T a b l e 1:** Averaged values of DSC coefficients for 2D U-Net and 3D U-Net models

Layer	2D U-Net	3D U-Net
Upper layer of stratum corneum	0.9	0.89
Ordered stratum corneum	0.94	0.94
Cellular layer of epidermis	0.89	0.87
Dermis	0.99	0.98

**Figure 9.**
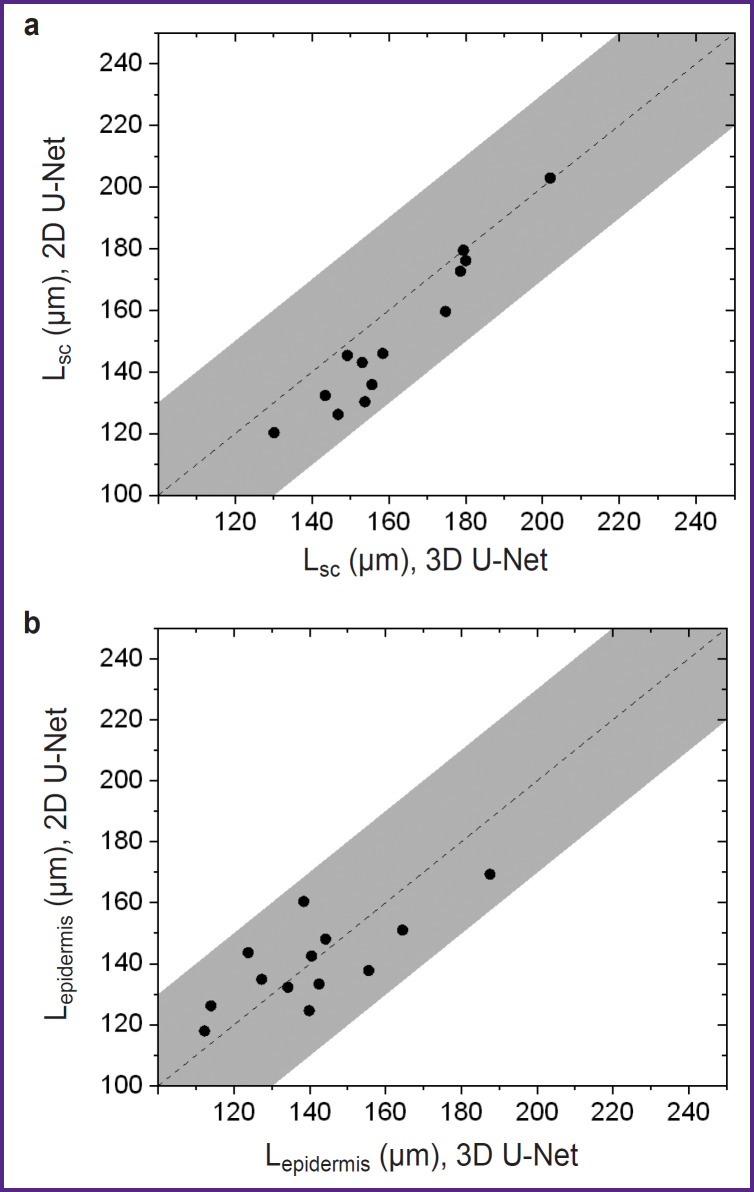
Comparison of the results of determining the thicknesses of the ordered stratum corneum (a) and the cellular layer of epidermis (b) for 2D U-Net and 3D U-Net models for layer segmentation in 3D OCT images from the test sample The gray band shows the possible error of boundary position estimation related to the axial spatial resolution of the OCT system

Further testing of the developed segmentation algorithms was performed on an additional set of 16 images of thick human skin (obtained from 8 volunteers) that did not overlap with the training and test samples. Segmentation was performed for all images in this sample, from which layer thicknesses were determined assuming that the average refractive index of the skin is 1.4. The results of comparing the thickness estimates of the ordered stratum corneum and epidermis are shown in Figure 9.

The averaged values of ordered stratum corneum and epidermis thicknesses obtained from the results of OCT image segmentation are presented in Table 2 in comparison with the values obtained earlier in [24].

**T a b l e 2:** Thicknesses of morphologic layers of thick skin obtained from segmentation data in comparison with the results of [24]

Layer	Thickness (μm), 2D U-Net	Thickness (μm), 3D U-Net	Thickness (μm), [24]
Ordered stratum corneum	153±24	163±19	175 [147; 210]
Cellular layer of epidermis	137±17	137±20	119 [112; 126]

N o t e. Data are presented as M±SD and Me [Q1; Q3].

## Discussion

The results of applying the segmentation algorithm for a single B-scan (see Figure 6) show that both networks provide segmentation consistent with the original labeling and can be used to estimate the average layer thickness. However, visually, the application of 3D U-Net (see Figure 6 (d)) appears more accurate in this example: this network more accurately conveys the features of the boundary between the stratum corneum and the epidermis, representing the presence of papillary patterns, whereas the segmentation results using 2D U-Net (see Figure 6 (c)) show a less accurate topography of the boundary associated with papillary patterns. It should be noted that the results obtained for the upper stratum corneum layer cannot be interpreted unambiguously. The tissue boundary has the highest signal level due to scattering from the surface, determined by the high refractive index mismatch as well as the random orientation of the surface scales of the stratum corneum. Moreover, the contact of the OCT probe surface with the skin surface is not always tight, which leads to the formation of air lacunae, the boundaries of which also are manifested by a high signal level. Since the longitudinal resolution of the OCT system is 15 μm, this value represents the error in determining the position of the boundary. However, its value is comparable to the thickness of the upper layers of the stratum corneum, which in the image are adjacent to the surface of the contact probe.

Evaluation of the segmentation accuracy using the Sørensen–Dice coefficient (see Figure 8) demonstrated that all obtained DSC values exceed 0.8, with values for 2D U-Net for the same layers not lower than those obtained using 3D U-Net. The averaged DSC values (see Table 1) demonstrate a value of at least 0.87, with the 2D U-Net accuracy for all layers proving to be at least as good as that of the 3D U-Net. Thus, the initial assumption that the 3D information contained in the 3D training blocks can improve segmentation accuracy has not been confirmed. Presumably, the situation will be different in the presence of volumetric macro heterogeneities of the skin (tumors, hair follicles, etc.), and the use of a 3D model will improve the prediction result.

When comparing the quality of the developed algorithm with the quality of the labeling algorithms described in other studies with OCT images, there is a problem of correct comparison of the results, since each study segment different structural layers in images of skin of different localizations. Since morphologically the most similar layer of thick and thin skin is the dermis, we compared the DSC value obtained for the dermis with the similar value of other studies that investigated thin skin. In [20], the DSC for the dermis was 0.96, which is almost identical to the results of [18], where the DSC for the dermis was 0.96±0.01. It should be noted that both of these works used neural networks based on U-Net architecture. Application of the algorithm developed in the present work provides DSC values for the dermis of 0.99 and 0.98 for 2D U-Net and 3D U-Net models, respectively, which is comparable to the results of other works.

The analysis of thickness estimates of skin structural layers demonstrated (see Figure 9) that 3D U-Net gives higher estimates of the thickness of the ordered stratum corneum (see Figure 9 (a)) compared to 2D U-Net. The discrepancy between the results of the two models for this layer does not exceed the error due to the spatial resolution of the OCT device. Similar results for the epidermis cellular layer are presented in Figure 9 (b), which shows that the 3D U-Net network gives higher estimates of layer thickness for cases of greater epidermis thickness, whereas the opposite trend is observed for smaller thickness values. It should be noted that for almost all cases, the mismatch between the two models also does not exceed the instrumental error induced by the axial spatial resolution of the system.

The analysis of the sample-averaged structural layer thicknesses (see Table 2) showed that the 2D U-Net model gives lower values for the average values compared to the 3D U-Net model as well, but their difference does not exceed either the standard deviation in the group or the error provided by the spatial resolution of the OCT system. The obtained values also agree well with the values demonstrated earlier in [24]: the ranges of values for the thicknesses of the ordered stratum corneum calculated on the basis of the segmentation data fit completely within the ranges published in that work on the basis of the analysis of a large sample, while for the cellular layer of the epidermis the intervals overlap significantly. This shows the promise of the proposed approach in extracting morphologic information from skin OCT imaging arrays.

In addition to diagnostic problems, the data obtained by automatic segmentation of OCT images can be used to build models of light propagation in biological tissues and signal formation in optical diagnostics systems [25], as well as for dosimetry tasks in photodynamic therapy [26].

## Conclusion

This paper demonstrates the capabilities of convolutional neural networks based on U-Net architecture in the task of segmentation of 3D OCT images of human thick skin. The main goal of the study was to compare different approaches to the selection of image blocks for training the neural network, which ultimately determines its structure. To the best of our knowledge, no such studies have been conducted previously.

It was shown that the models show similar results in segmentation performance: DSC for the 2D U-Net model amounts to 0.90, 0.94, 0.89, 0.99 for the upper stratum corneum, ordered stratum corneum, cellular layer of epidermis, and dermis, respectively. These values for the 3D U-Net model amount to 0.90, 0.95, 0.88, 0.98. Hence, it is advisable to use the model for which it is easier to collect data for training. For example, for thin skin segmentation, it may be easier to collect a set of labeled 2D images. In the considered case, a semiautomatic initial labeling method was applied using a priori information about a particular 3D array, which allowed training the 3D model.

In most of the known works dealing with the segmentation of OCT images of skin, thin skin served as the object of study, whereas in the present work thick skin, which is morphologically different from thin skin, was investigated. The obtained estimates of thickness of the ordered stratum corneum and epidermal cellular layer are 153±24 and 137±17 μm, respectively, when using 2D U-Net data and 163±19 and 137±20 μm, respectively, for 3D U-Net data.

The software with the embedded proposed models can be an important addition to the OCT system and can be applied directly in clinical practice.
